# On a New Means of Rendering Surgical Operations Painless

**Published:** 1847-01

**Authors:** 


### ON A NEW MEANS OF RENDERING SURGICAL OPERATIONS PAINLESS.

Just as our last proof was passing through our hands, we received from our medical friends in Boston the account of a matter so interesting to surgeons, and indeed to every one, that we take the opportunity of introducing it here. We know nothing more of this new method of eschewing pain than what is contained in the following extracts from two private letters, kindly written to us by our excellent friends, Dr. Ware and Dr. Warren, of Boston—both men of the highest eminence in their profession in America—and, we may truly say, in Europe also. It is impossible however, not to regard the discovery as one of the very highest importance, not in the practice of operative surgery only, but also, as Dr. Ware suggests, in practical medicine also. We trust our friends will forgive us for putting into print their private communications. The importance of the subject and the necessity of authenticating the statements, are our excuses. The authors of the discovery are Dr. C. T. Jackson and Dr. Morton.

"Boston, November 29, 1846.

"I found, on my arrival here, a new thing in the medical world, or rather the new application of an old thing, of which I think you will like to hear. It is a mode of rendering patients insensible to the pain of surgical operations, by the inhalation of the vapour of the strongest sulphuric ether. They are thrown into a state nearly resembling that of complete intoxication from ardent spirits or of narcotism from opium. This state continues but a few minutes—five to ten—but, during it, the patient is insensible to pain. A thigh has been amputated, a breast extirpated, teeth drawn, without the slightest suffering. The number of operations of various kinds, especially those in dentistry, has been very considerable, and I believe but few persons resist the influence of the agent.

"The effect is not exactly the same on all. In some, the insensibility is entire, and the patient is aware of nothing which is going on; in others, a certain degree of the power of perception remains, the patient knows what the operator is doing, perceives him for example, take hold of a tooth and draw it out, feels the grating of the instrument, but still has no pain.

"There are no subsequent ill effects to detract from the value of this practice, none even so great as those which follow a common dose of opium. One person told me she had some unpleasant sensations in the head for a short time, and was weak, languid, and faintish through the day, but not more so than she ordinarily was from having a tooth drawn. Another told me that he experienced something of the same kind and in addition that his breath smelt very strongly of *ether* for forty-eight hours, and was indeed so strongly impregnated with it as to affect the air of the room in which he sat, so as to be disagreeable to others.

"One of our best operative surgeons informs me that he regards it as chiefly applicable to cases of the large and painful operations which are performed rapidly, and do not require any very nice dissection, but that for the more delicate operations, which require some time, he would prefer to have the patient in his usual state. But it is impossible at present to judge what will be the limits to the application of such an agent. Objections may arise of which we do not dream, and evils may be found to follow, which we do not now perceive. Still it certainly promises much in surgery, and perhaps may be capable of application for other purposes beside the alleviation of pain. Would it not be worthy of trial in tetanus, in asthma, and in various cases of violent internal pain, especially from supposed spasms?

"It was brought into use by a dentist, and is now chiefly employed by that class of practitioners. He has taken out a patent for the discovery, and has despatched persons to Europe to secure one there also; so you will soon hear of it, and probably have an opportunity of witnessing its effects. Faithfully yours, John Ware."

"Boston, November 24th, 1846.

"You may have heard of the respiration of ether to prevent pain in surgical operations. In six cases I have had it applied with satisfactory success and no unpleasant sequel. "I remain, &c., John C. Warren."

Since the above was in type we have seen a more extended communication on the same subject, published by Dr. Bigelow in the 'Boston Medical and Surgical Journal.' The more material parts of this we hasten to extract:

"It remains briefly to describe the process of inhalation by the new method, and to state some of its effects. A small two-necked glass globe contains the prepared vapour, with sponges to enlarge the evaporating surface. One aperture admits the air to the interior of the globe, whence, charged with vapour, it is drawn through the second into the lungs. The inspired air thus passes through the bottle, but the expiration is diverted by a valve in the mouth-piece, and escaping into the apartment, is thus prevented from vitiating the medicated vapour.

"A boy of 16, of medium stature and strength, was seated in the chair. The first few inhalations occasioned a quick cough, which afterwards subsided; at the end of eight minutes the head fell back, and the arms dropped, but owing to some resistance in opening the mouth, the tooth could not be reached before he awoke. He again inhaled for two minutes, and slept three minutes, during which time the tooth, an inferior molar, was extracted. At the moment of extraction the features assumed an expression of pain, and the hand was raised. Upon coming to himself he said he had had a 'first-rate dream—very quiet,—'and had dreamed of Napoleon—had not the slightest consciousness of pain—the time had seemed long;' and he left the chair feeling no uneasiness of any kind, and evidently in a high state of admiration. The pupils were dilated during the state of unconsciousness, and the pulse rose from 130 to 142.

"A girl of 16 immediately occupied the chair. After coughing a little, she inhaled during three minutes, and fell asleep, when a molar tooth was extracted, after which she continued to slumber tranquilly during three minutes more. At the moment when force was applied she flinched and frowned, raising her hand to her mouth, but she said she had been dreaming a 'pleasant dream, and knew nothing of the operation.

"A stout boy of 12 at the first inspiration coughed considerably, and required a good deal of encouragement to induce him to go on. At the end of three minutes from the first fair inhalation, the muscles were relaxed and the pupil dilated. During the attempt to force open the mouth he recovered his consciousness, and again inhaled during two minutes, and in the ensuing one minute two teeth were extracted, the patient seeming somewhat conscious, but upon actually awaking, he declared 'it was the best fun he ever saw,' avowed his intention to come there again, and insisted upon having another tooth extracted upon the spot. A splinter which had been left afforded an opportunity of complying with his wish, but the pain proved to be considerable. Pulse at first 110, during sleep 96, afterward 144, pupils dilated.

"These cases, which occurred successively in about an hour, at the room of Dr. Morton, are fair examples of the average results produced by the inhalation of the vapour, and will convey an idea of the feelings and expressions of many of the patients subjected to the process.

"The inhalation, after the first irritation has subsided, is easy, and produces a complete unconsciousness at the expiration of a period varying from two to five or six, sometimes eight minutes; its duration varying from two to five minutes, during which the patient is completely insensible to the ordinary tests of pain. The pupils, in the cases I have observed, have been generally dilated; but with allowance for excitement and other disturbing influences, the pulse is not affected, at least in frequency. The patient remains in a calm and tranquil slumber, and wakes with a pleasurable feeling.

"It is natural to inquire whether no accidents have attended the employment of a method so wide in its application, and so striking in its results. I have been unable to learn that any serious consequences have ensued. One or two robust patients have failed to be affected. I may mention, as an early and unsuccessful case, its administration in an operation performed by Dr. Hayward, where an elderly woman was mdae to inhale the vapour for at least half an hour without effect. Though I was unable at the time to detect any imperfection in the process, I am inclined to think that such existed. One woman became much excited, and required to be confined to the chair. As this occurred to the same patient twice, and in no other case, as far as I have been able to learn, it was evidently owing to a peculiar susceptibility. Very young subjects are affected with nausea and vomiting, and for this reason Dr. Morton has refused to administer it to children. Finally, in a few cases, the patient has continued to sleep tranquilly for eight or ten minutes, and once, after a protracted inhalation, for the period of an hour.

"The following case, which occurred a few days since, will illustrate the probable character of future accidents. A young man was made to inhale the vapour while an operation of limited extent, but somewhat protracted duration, was performed by Dr. Dix upon the tissues near the eye. After a good deal of coughing, the patient succeeded in inhaling the vapour, and fell asleep at the end of about ten minutes. During the succeeding two minutes the first incision was made, and the patient awoke, but unconscious of pain. Desiring to be again inebriated, the tube was placed in his mouth, and retained there about twenty-five minutes. the patient being apparently half affected, but, as he subsequently stated, unconscious. Respiration was performed partly through the tube and partly with the mouth open. Thirty-five minutes had now elapsed, when I found the pulse suddenly diminishing in force, so much so that I suggested the propriety of desisting. The pulse continued decreasing in force, and from 120 had fallen to 96. The respiration was very slow, the hands cold, and the patient insensible. Attention was now, of course, directed to the return of respiration and circulation. Cold affusions, as directed for poisoning with alcohol, were applied to the head, the ears were syringed, and ammonia presented to the nostrils and administered internally. For fifteen minutes the symptoms remained stationary, when it was proposed to use active exercise, as in a state of narcotism from opium. Being lifted to his feet, the patient soon made an effort to move his limbs, and the pulse became more full, but again decreased in the sitting posture, and it was only after being compelled to walk during half an hour that the patient was able to lift his head. Complete consciousness returned only at the expiration of an hour. In this case the blood was flowing from the head, and rendered additional loss of blood unnecessary. Indeed the probable hemorrhage was previously relied on as salutary in its tendency.

"Two recent cases serve to confirm, and one I think to decide, the great utility of this process. On Saturday, the 7th November, at the Massachusetts General Hospital, the right leg of a young girl was amputated above the knee, by Dr. Hayward, for disease of this joint. Being made to inhale the preparation, after protesting her inability to do so from the pungency of the vapour, she became insensible in about five minutes. The last circumstance she was able to recal was the adjustment of the mouth-piece of the apparatus, after which she was unconscious until she heard some remark at the time of securing the vessels—one of the last steps of the operation. Of the incision she knew nothing, and was unable to say, upon my asking her, whether or not the limb had been removed. She refused to answer several questions during the operation, and was evidently completely insensible to pain or other external influences. This operation was followed by another, consisting of the removal of a part of the lower jaw by Dr. Warren. The patient was insensible to the pain of the first incision, though she recovered her consciousness in the course of a few minutes.

"I am as yet unable to generalize certain other symptoms to which I have directed attention. The pulse has been, as far as my observation extends, unaltered in frequency, though somewhat diminished in volume, but the excitement preceding an operation has, in almost every instance, so accelerated the pulse that it has continued rapid for a length of time. The pupils are in a majority of cases dilated; yet they are in certain cases unaltered, as in the above case of amputation.

"The duration of the insensibility is another important element in the process.

When the apparatus is withdrawn at the moment of unconsciousness, it continues upon the average two or three minutes, and the patient then recovers completely or incompletely, without subsequent ill effects.

"But if the respiration of the vapour be prolonged much beyond the first period, the symptoms are more permanent in their character. In one of the first cases, that of a young boy, the inhalation was continued during the greater part of ten minutes, and the subsequent narcotism and drowsiness lasted more than an hour. In a case alluded to before, the narcotism was complete more than twenty minutes; the insensibility approached to coma.

"The process is obviously adapted to operations which are brief in their duration, whatever be their severity. Of these the two most striking are, perhaps, amputations and the extraction of teeth. In protracted dissections, the pain of the first incision alone is of sufficient importance to induce its use; and it may hereafter produce a narcotism of an hour's duration. It is not unlikely to be applicable in cases requiring a suspension of muscular action; such as the reduction of dislocations or of strangulated hernia; and, finally, it may be employed in the alleviation of functional pain, of muscular spasm, as in cramp and colic, and as a sedative or narcotic."

---

*P.S. Dec.* 22.—Yesterday we had ourselves the satisfaction of seeing this new mode of cheating pain put in practice by a master of chirurgery on our own side of the Atlantic. In the theatre of University College Hospital, Mr. Liston amputated the thigh of a man previously narcotised by inhalation of the ether vapour. Shortly after being placed on the operating table, the patient began to inhale, and became apparently insensible in the course of two or three minutes. The operation then commenced; and the limb was removed in what seemed to us a marvellously short space of time—certainly less than a minute; the patient remaining, during the incisions and the tying of the arteries, perfectly still and motionless. While the vessels were being secured, on being spoken to he roused partially up (still showing no signs of pain), and answered questions put to him in a slow, drowsy manner. He declared to us that at no part of the operation had he felt *pain*, though he seemed to be partially conscious; he heard some words, and felt that *something* was being done to his limb. He was not aware, till told, that the limb was off, and, when he knew it, expressed great gratification at having been saved from pain. The man seemed quite awake when removed from the operation-room, and continued so. Everything has since proceeded as usual, and very favorably.

Mr. Liston afterwards performed one of the minor but most painful operations of surgery—the partial removal of the nail in *onychia*—on a man similarly narcotised, and with precisely the same result. The patient seemed to feel no pain, and, upon rousing up after the operation, declared that he had felt none.

In these cases the ether vapour was administered by means of an ingenious apparatus extemporaneously contrived by Mr. Squire, of Oxford street. It consisted of the *bottom part* of a Nooth's apparatus, having a glass funnel filled with sponge soaked in pure *washed* ether, in the upper orifice, and one of Read's flexible inhaling tubes in the lower. As the ether fell through the neck of the funnel, it became vaporized, and the vapour being heavy, descended to the bottom of the vase, and was thence inspired through the flexible tube. No heat was applied to the apparatus or the ether.

The momentous details given above suggest many remarks which we have no room to record. We are only able to observe that if the new process shall supersede that employed, with a like object, by the mesmerists, we must concede to them that it supplies, from analogy, additional reasons for believing in their statements in regard to the production by their process, of insensibility to pain. (See Art. X, in our last Number.) The readers of two articles in our last and present Numbers, will also observe the bearing of some of the present results on the semi-psychical discussions contained in them. J. F.

